# Testing Patterns for CKD-MBD Abnormalities in a Sample US Population

**DOI:** 10.1016/j.ekir.2020.12.036

**Published:** 2021-01-06

**Authors:** James B. Wetmore, Yuanyuan Ji, Akhtar Ashfaq, David T. Gilbertson, Nicholas S. Roetker

**Affiliations:** 1 Chronic Disease Research Group, Hennepin Healthcare Research Institute, Minneapolis, Minnesota, USA; 2 Division of Nephrology, Hennepin County Medical Center and Department of Medicine, University of Minnesota, Minneapolis, Minnesota, USA; 3 OPKO Pharmaceuticals, Miami, Florida

**Keywords:** chronic kidney disease, mineral/bone disorder, parathyroid hormone, phosphorus, 25D

## Abstract

**Introduction:**

Patterns of testing, treatment, and retesting following treatment for disorders of chronic kidney disease mineral bone disorder (CKD-MBD) have not been explored using a large electronic database.

**Methods:**

To determine concordance with CKD-MBD management guidelines, we used 2010 to 2019 data from an electronic health record (>50 million patients) to create cohorts of incident CKD stage 3, 4, and 5 patients using diagnosis codes and estimated glomerular filtration rates. The CKD-MBD test ordering and relevant drug prescribing were assessed during follow-up. We estimated cumulative incidence of posttreatment retesting (death as competing risk). We used multivariable Cox regression to examine baseline characteristics and pretreatment test results as predictors of retesting.

**Results:**

For 215,553 stage 3, 43,576 stage 4, and 11,407 stage 5 CKD patients, the mean follow-up was 2.3, 1.7, and 0.6 years, respectively. Only 46% of stage 4 and 41% of stage 5 patients underwent parathyroid hormone (PTH) testing, 74% and 73% had phosphorus testing, and 38% and 25% had 25D testing. By 1 year after vitamin D sterol treatment, only 50%, 53%, and 60% of stage 3, 4, and 5 patients had been retested for PTH. By 1 year after treatment with ergocalciferol or cholecalciferol, only 46%, 49%, and 55% had 25D reassessed. Pretreatment levels of PTH and 25D were not associated in a graded fashion with likelihood of retesting after treatment. Rates of retesting were not highest for patients with the highest and lowest pre-treatment PTH and 25D levels, respectively.

**Conclusion:**

Frequency of testing for CKD-MBD abnormalities and posttreatment retesting appears to be suboptimal.

## Graphical abstract

**Figure:**
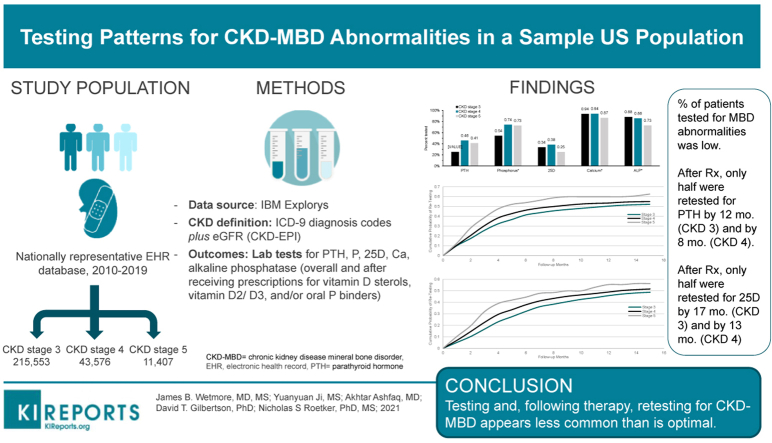

Chronic kidney disease−mineral and bone disorder (CKD-MBD) is a term encompassing a constellation of disorders related to mineral metabolic analytes, alterations in bone physiology, and presence of vascular calcification.^1^ It becomes increasingly common as kidney function declines,^2^ and is associated with adverse outcomes such as cardiovascular events and fractures.^1^^,^^3^ As such, organizations such as KDIGO (Kidney Disease: Improving Global Outcomes) provide guidelines for its diagnosis and treatment.^3^

We recently reported relatively low rates of testing for parathyroid hormone (PTH), 25-hydroxyvitamin D (25D), and phosphorus in a US population of Medicare beneficiaries, suggesting that physicians may be under-testing patients for these important abnormalities relative to the guideline recommendations^4^; this finding is concordant with other studies examining testing rates, particularly of PTH.^5^, ^6^, ^7^, ^8^, ^9^, ^10^, ^11^ However, in our previous work, we lacked access to patient-level laboratory data. Accordingly, to further explore how patients are being tested and treated for CKD-MBD, we constructed a patient cohort from IBM Explorys, a large electronic health record (EHR) database. This patient-level database contains clinical diagnoses, as can be found in many administrative claims databases, as well as laboratory data and prescription records for CKD-MBD−related medications such as vitamin D sterols, 25D (also known colloquially as “nutritional vitamin D”), and oral phosphate binders. We specifically examined rates of testing for CKD-MBD abnormalities, distribution of laboratory abnormalities upon testing, rates of treatment, and rates of retesting after therapy initiation. We predicted that rates of initial testing would be relatively low, and that rates of retesting, even after therapy was initiated, would be suboptimal. If so, this would suggest a care gap, both in initial detection of abnormalities and in appropriate long-term management of CKD-MBD.

## Materials and Methods

### Study Design and Data Sources

We used data from 2010 to 2019 from IBM Explorys, a large EHR database, to conduct a retrospective cohort analysis. IBM Explorys aggregates data from 26 US health care networks encompassing 360 hospitals and more than 50 million patients, representing approximately 15% of the US population. These data included patient-level information on demographics and health care encounters (office, outpatient hospital, emergency department, inpatient hospital, etc.), including records of diagnoses, procedures, laboratory tests, and prescribed drugs. These data were coded using Systematized Nomenclature of Medicine Clinical Terms (SNOMED CT), International Classification of Diseases, Ninth/Tenth Edition, Clinical Modification (ICD-9/10-CM) codes, and Current Procedural Terminology (CPT) codes for diagnoses and/or procedures; Logical Information Identifiers Names and Codes (LOINC) for laboratory test results; and RxNorm for prescribed medications.

### CKD Cohorts

We identified 3 cohorts, consisting of patients with incident CKD stages 3, 4, and 5 disease, respectively. Inclusion in each cohort was determined using clinical diagnosis codes for CKD stage 3, 4, or 5 and a confirmatory estimated glomerular filtration rate (eGFR) laboratory value. Codes used to define the cohorts are shown in Supplementary Table S1. Separately for each CKD cohort, we first identified the earliest diagnosis of the respective CKD stage occurring between January 2011 and March 2019. The diagnosis date was defined as the index date for that stage. Next, we identified the eGFR value occurring closest in time, within 60 days before or on the index date. If this eGFR value was outside the range of the CKD stage diagnosis, or if there was no eGFR determination, the patient was excluded. Then, patients with a diagnosis of a more advanced CKD stage or with end-stage renal disease (ESRD) or a kidney transplant before the index date were excluded. Patients younger than 18 years of age, who had no health care encounters after the index date, or who died on the index date were also excluded. Finally, to ensure adequate availability of baseline EHR data, we excluded patients without at least 1 health care encounter occurring 1 year or more before the index date. Patients in each CKD cohort were followed from the index date until the earliest of the following: the index date for a more advanced CKD stage, ESRD or kidney transplantation, death, a 1-year period without a health care encounter, or March 31, 2019.

### Laboratory Testing, Test Results, and Drug Prescribing

We identified laboratory test ordering and results for the biochemical markers PTH, phosphorus, 25D, calcium, and alkaline phosphatase (ALP) in the EHR using the codes shown in Supplementary Table S2. Test orders included codes for stand-alone tests for each biochemical marker and for various testing panels (e.g., metabolic, renal, and hepatic function) that included phosphorus, calcium, or ALP as component tests. Testing was defined by at least 1 code for a laboratory test order or test result during follow-up. Test results with values outside of the plausible range, as described in Supplementary Table S2, were excluded. We also identified prescriptions for vitamin D sterols (e.g., calcitriol, paricalcitol, and doxercalciferol), vitamin D_2_ and D_3_ (ergocalciferol and cholecalciferol), and oral phosphate binders using the codes shown in Supplementary Table S3. Treatment was defined by at least 1 prescription code during follow-up.

### Covariates

Assessed demographic information included age, sex, and race. Other clinical characteristics, including number of prior hospitalizations and comorbid conditions, were assessed during the baseline period, a 1-year period preceding the index date. Each comorbid condition was defined by the presence of a corresponding diagnosis code on at least 1 inpatient hospital or observation stay encounter claim or at least 2 encounter claims of any other type (e.g., outpatient) separated by at least 30 days. The diagnosis codes used for each condition, most of which were components of the Charlson or Elixhauser algorithms,^12^ are listed in Supplementary Table S4.

### Statistical Analysis

Analyses were performed separately for each of the CKD stage 3, 4, and 5 cohorts. Descriptive statistics (frequencies and percentages) are presented for baseline characteristics. Patterns of laboratory test ordering and drug prescribing were described using percentages and rates, per 100 person-years, during the follow-up period. The distributions of laboratory test results were described using percentages of patients exceeding clinically relevant thresholds, density plots (a smoothed version of a histogram), and descriptive statistics. We estimated the cumulative incidence of laboratory retesting following treatment, treating death as a competing risk event. We used multivariable Cox proportional hazard regression to examine baseline characteristics and pretreatment test result values as predictors of laboratory retesting; regression models for CKD stage 4 and 5 patients were excluded because of the small numbers of patients. For all analyses involving laboratory test ordering or test results for PTH, phosphorus, and 25D, we excluded patients with a history of treatment with vitamin D sterols, ergocalciferol/cholecalciferol, and oral phosphate binders, respectively. Likewise, when assessing laboratory retesting following treatment, we excluded patients with a history of treatment (before the index date) or without a laboratory test result before the current treatment.

## Results

**Figure 1:**
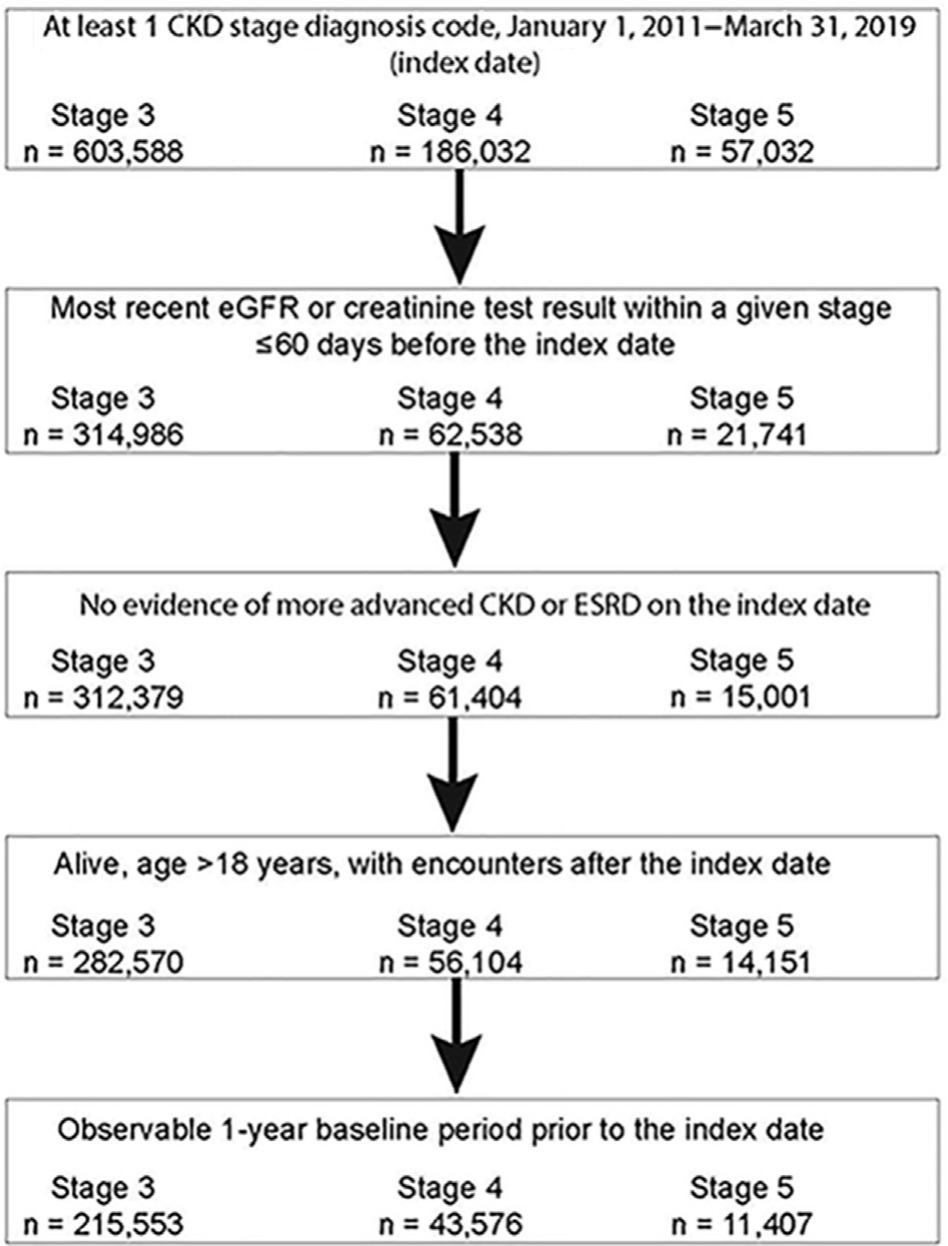
Construction of the study cohort.

Construction of the study cohorts is shown in Figure 1. After exclusions, 215,553 patients with stage 3 CKD, 43,576 patients with stage 4 CKD, and 11,407 patients with stage 5 CKD were identified. Mean follow-up was 2.3, 1.7, and 0.6 years, respectively; more detail is shown in Supplementary Table S5.

Baseline characteristics of each of the 3 CKD cohorts are shown in Table 1. Mean ages were 72.6, 72.3, and 65.3 years for the CKD 3, 4, and 5 cohorts, respectively; 50.8%, 46.0%, and 50.0% of patients, respectively, were men. White patients comprised 84.2% of the CKD 3 cohort and 80.2% of the CKD 4 cohort, but only 67.0% of the CKD 5 cohort. Across the stages, approximately 42% of patients had been hospitalized in the previous year. Percentages with diabetes were 37.7%, 48.2%, and 53.6% in stages 3, 4, and 5, respectively; corresponding percentages for hypertension were 68.5%, 74.3%, and 79.8%.

**Table 1:** Patient characteristics in the CKD stage 3, 4, and 5 cohorts

Characteristic	CKD stage 3 n = 215,553	CKD stage 4 n = 43,576	CKD stage 5 n = 11,407
	n (%)	n (%)	n (%)
Age, mean (SD)	72.61 (11.53)	72.30 (12.64)	65.34 (14.45)
Age group, yr			
18–40	3168 (1.47)	1111 (2.55)	756 (6.63)
41–60	30,595 (14.19)	6,625 (15.20)	3,313 (29.04)
61–70	50,920 (23.62)	9221 (21.16)	2945 (25.82)
70–80	66,257 (30.74)	12,571 (28.85)	2533 (22.21)
≥81	64,613 (29.98)	14,048 (32.24)	1860 (16.31)
Sex			
Male	109,441 (50.77)	20,044 (46.00)	5698 (49.95)
Female	106,112 (49.23)	23,532 (54.00)	5709 (50.05)
Race			
White	181,398 (84.15)	34,925 (80.15)	7646 (67.03)
Black	28,273 (13.12)	6969 (15.99)	3102 (27.19)
Other	5882 (2.73)	1682 (3.86)	659 (5.78)
Hospitalization during 1-yr baseline			
Yes	91,261 (42.34)	18,656 (42.81)	4813 (42.19)
No	124,292 (57.66)	24,920 (57.19)	6594 (57.81)
Comorbid conditions during 1-yr baseline			
Atherosclerotic heart disease	52,052 (24.15)	11,307 (25.95)	2579 (22.61)
Congestive heart failure	45,244 (20.99)	13,867 (31.82)	3361 (29.46)
Cardiac arrhythmia	55,115 (25.57)	12,328 (28.29)	2372 (20.79)
Cerebrovascular disease	20,503 (9.51)	4545 (10.43)	1092 (9.57)
Peripheral vascular disease	24,290 (11.27)	6064 (13.92)	1537 (13.47)
Chronic obstructive pulmonary disease	42,170 (19.56)	9321 (21.39)	2122 (18.60)
Gastrointestinal bleeding	5419 (2.51)	1362 (3.13)	428 (3.75)
Liver disease	8603 (3.99)	2103 (4.83)	661 (5.79)
Cancer	21,941 (10.18)	4679 (10.74)	1107 (9.70)
Diabetes	81,238 (37.69)	20,988 (48.16)	6113 (53.59)
Hypertension	147,639 (68.49)	32,365 (74.27)	9105 (79.82)
Dementia	5908 (2.74)	1189 (2.73)	203 (1.78)
HIV	687 (0.32)	137 (0.31)	74 (0.65)

CKD, chronic kidney disease.

**Figure 2:**
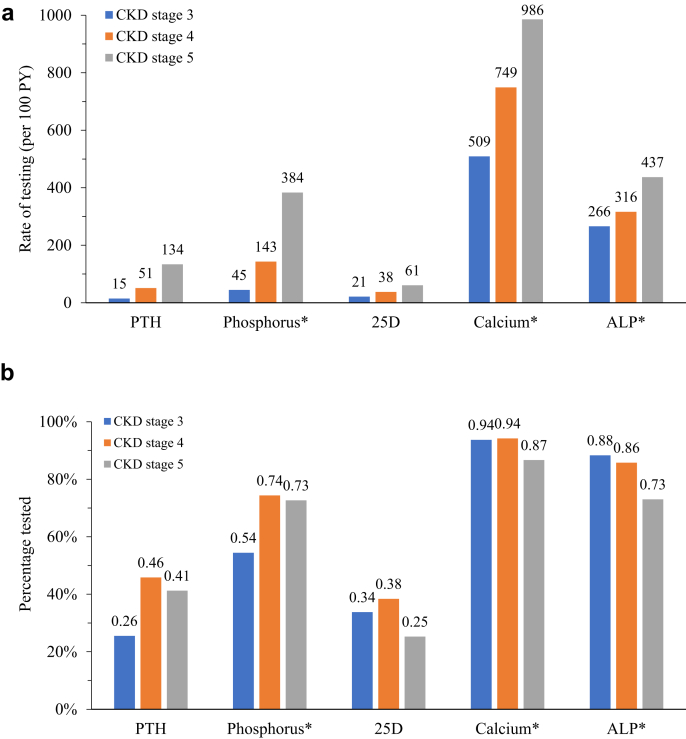
Patterns of test ordering for biochemical markers of CKD-MBD. (a) Rates per 100 person-years; (b) percentages. ALP, alkaline phosphatase; CKD, chronic kidney disease; MBD, mineral bone disorder; PTH, parathyroid hormone; PY, person-years.

Patterns of test ordering for biochemical markers of CKD-MBD are shown in Figure 2. Patterns are shown using percentages and, because length of follow-up time varied substantially, rates in person-years. Rates of test ordering increased for each analyte as CKD stage worsened. However, percentages of test ordering for PTH, phosphorus, and 25D were substantially less than 100%. Among patients with stage 3 CKD, 26% underwent a test for PTH, 34% for 25D, and 54% for phosphorus. However, only 46% of stage 4 patients and 41% of stage 5 patients underwent a PTH test; only 74% and 73%, respectively, a test for phosphorus; and only 38% and 25%, respectively, a test for 25D.

**Figure 3:**
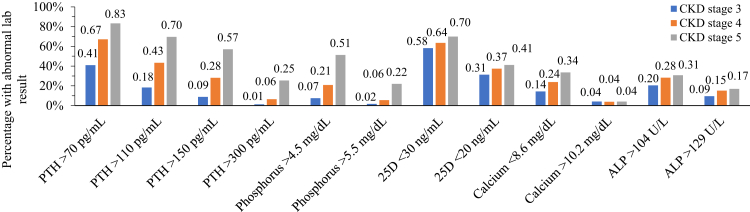
Prevalence of abnormal results for each analyte, in the absence of specific treatment and separately by stage of CKD. ALP, alkaline phosphatase; CKD, chronic kidney disease; lab, laboratory; PTH, parathyroid hormone.

Prevalence of abnormal results for each analyte, in the absence of specific treatment and separately by stage of CKD, is shown in Figure 3. Abnormal levels are illustrated using 4 thresholds for elevated PTH, 2 each for elevated phosphorus and ALP, 2 for 25D (deficiency and insufficiency), and 2 for calcium (hypo- and hypercalcemia). Notably, in the absence of treatment with a vitamin D sterol, 18% of stage 3 patients had a PTH level >110 pg/ml, with 9% >150 pg/ml; only 17% of stage 5 patients had a PTH ≤70 pg/ml. Of stage 3 patients, 14% had a calcium level ≤8.6 mg/dl and 9% had an ALP level >129 U/L. Density plots displaying the full distribution of laboratory results for each biochemical marker are shown in Supplementary Figure S1*.*

**Figure 4:**
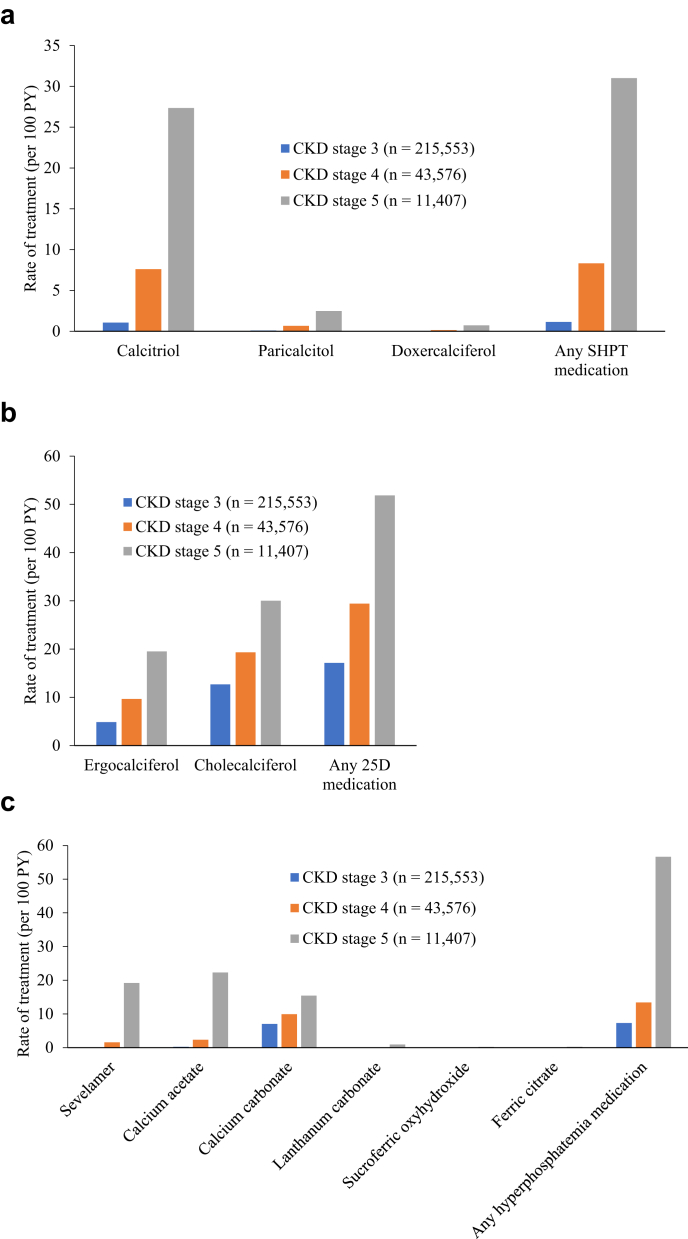
Patterns in treatment rates. (a) SHPT medications; (b) 25D medications; (c) phosphate binders. CKD, chronic kidney disease; PY, person-years; SHPT, secondary hyperparathyroidism.

Patterns in treatment rates are shown in Figure 4. For vitamin D sterols, calcitriol was, by far, the most common drug used; overall treatment rates increased roughly 7-fold from stage 3 to stage 4, and by about 4-fold from stage 4 to stage 5. Cholecalciferol was used more often than ergocalciferol throughout, but the ratio of cholecalciferol to ergocalciferol users decreased steadily as CKD stage worsened (from about 3:1 at stage 3 to 1.5:1 at stage 5). Among the oral phosphate binders, only in stage 5 patients were calcium acetate and sevelamer used more often than calcium carbonate; the latter may have been prescribed for reasons other than treating hyperphosphatemia.

The distributions of analyte levels measured closest in time before treatment initiation are shown in Table 2, by CKD stage. Mean levels of PTH before treatment in stages 3, 4, and 5 CKD were 154, 221, and 352 pg/ml, respectively; mean levels of 25D before treatment ranged from 24.3 ng/ml (stage 3) to 20.9 ng/ml (stage 5). Mean levels of phosphorus before treatment were 4.6 mg/dl in stage 4 and 5.8 mg/dl in stage 5.

**Table 2:** Distribution of laboratory test results before first treatment in the CKD stage 3, 4, and 5 cohorts

	Patients (n)^b^	Distribution of most recent laboratory test result before treatment^a^						
		Mean	SD	Min	Q1	Median	Q3	Max
Distribution of PTH (pg/ml) before first treatment with activated vitamin D compounds								
Stage 3	2163	153.7	115.8	1.0	90.5	125.0	185.0	1528
Stage 4	2016	221.0	164.5	3.0	128.0	182.0	267.0	2541
Stage 5	379	351.8	261.0	10.0	167.0	283.0	446.1	2072
Distribution of 25D (ng/ml) before first treatment with nutritional vitamin D (25D)								
Stage 3	12,422	24.3	14.0	1.0	14.0	22.0	31.0	150
Stage 4	2620	22.4	13.3	1.0	12.8	20.0	29.0	110.2
Stage 5	286	20.9	13.1	2.6	11.8	18.0	27.0	75.0
Distribution of phosphorus (mg/dl) before first treatment with oral phosphate binder								
Stage 3	8042	3.6	1.2	1.5	2.9	3.4	4.0	13.2
Stage 4	3026	4.6	1.6	1.5	3.5	4.3	5.6	13.7
Stage 5	831	5.8	1.6	1.6	4.8	5.7	6.6	15.7

CKD, chronic kidney disease; Max, maximum; Min, minimum; PTH, parathyroid hormone.

a Among treatment-naive patients.

b Distributions were calculated among patients who had no prior history of treatment (i.e., before the index date of the given CKD stage). Laboratory test results were considered only if the test occurred on or after the index date and before or on the date of first treatment.

**Figure 5:**
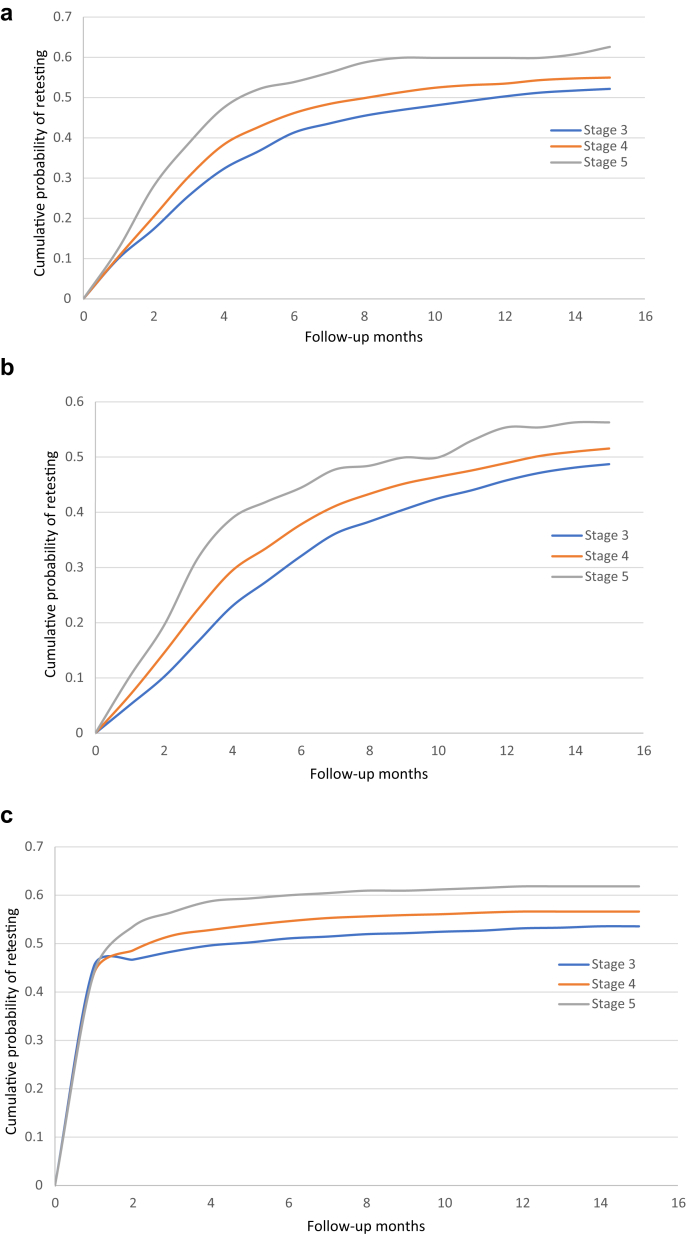
Cumulative probability of analyte retesting following initiation of (a) SHPT medication, (b) 25D medication, or (c) phosphate binders. CKD, chronic kidney disease; SHPT, secondary hyperparathyroidism.

The cumulative probability of analyte retesting following therapy initiation is shown in Figure 5. Following treatment with vitamin D sterols, the time point by which half of the patients had undergone retesting for PTH levels was 12 months, 8 months, and 4.5 months for patients with CKD stages 3, 4, and 5, respectively. Analogously, half of the patients underwent retesting for 25D levels by 17 months (stage 3), 13 months (stage 4), and 10 months (stage 5) after treatment with cholecalciferol or ergocalciferol.

Hazard ratios from multivariable Cox regression models of factors associated with retesting of PTH, 25D, and phosphorus following therapy initiation are shown in Table 3 for CKD stage 3. Age, sex, and race were generally not associated with analyte retesting, except that female sex appeared to be associated with a lower rate of phosphorus retesting. History of hospitalization in the previous year was associated with a lower rate of retesting for PTH and 25D. Liver disease was associated with an increased rate of PTH retesting, and dementia with a decreased rate of 25D retesting. Several comorbid conditions, including heart failure, peripheral vascular disease, chronic obstructive pulmonary disease, gastrointestinal bleeding, and liver disease, were associated with an increased rate of phosphorus retesting. Despite some indication that greater pretreatment levels of PTH and 25D were associated with an increased rate of retesting for these analytes, clear dose−response relationships were lacking. Only in the case of phosphorus did rates of retesting appear to increase in a dose−response fashion according to the pretreatment phosphorus level.

**Table 3:** Multivariable Cox proportional hazard regression for factors associated with retesting after treatment with activated vitamin D compounds, nutritional vitamin D, or phosphate binders in CKD stage 3 patients

	Hazard ratio (95% confidence interval)		
	PTH	25D	Phosphorus
	n = 2,163	n = 12,422	n = 8,042
Age group, yr			
18–40	1 (Ref)	1 (Ref)	1 (Ref)
41–60	1.04 (0.62–1.74)	1.05 (0.88–1.26)	0.97 (0.79–1.19)
61–70	1.10 (0.66–1.83)	1.11 (0.93–1.33)	1.01 (0.83–1.24)
70–80	0.98 (0.59–1.62)	1.04 (0.87–1.25)	0.95 (0.77–1.17)
≥81	0.86 (0.51–1.43)	0.88 (0.73–1.06)	0.96 (0.78–1.19)
Female sex	1.01 (0.90–1.13)	0.99 (0.94–1.04)	0.85 (0.81–0.90)
Race			
White	1 (Ref)	1 (Ref)	1 (Ref)
Black	1.07 (0.93–1.23)	0.99 (0.93–1.05)	1.03 (0.95–1.12)
Other	0.90 (0.58–1.39)	1.03 (0.89–1.19)	1.05 (0.87–1.26)
Hospitalization^a^	0.83 (0.73–0.95)	0.89 (0.84–0.94)	1.02 (0.96–1.08)
Comorbid conditions^a^			
Atherosclerotic heart disease	1.06 (0.91–1.23)	1.04 (0.97–1.11)	1.00 (0.93–1.07)
Congestive heart failure	1.05 (0.89–1.23)	0.99 (0.92–1.07)	1.15 (1.07–1.24)
Cardiac arrhythmia	1.02 (0.88–1.18)	0.97 (0.91–1.04)	1.05 (0.98–1.13)
Cerebrovascular disease	0.86 (0.69–1.06)	0.97 (0.88–1.06)	1.01 (0.92–1.10)
Peripheral vascular disease	1.03 (0.85–1.25)	0.94 (0.86–1.02)	1.16 (1.07–1.26)
COPD	0.91 (0.78–1.06)	0.95 (0.89–1.02)	1.09 (1.01–1.16)
Gastrointestinal bleeding	0.94 (0.61–1.46)	0.97 (0.79–1.19)	1.23 (1.06–1.42)
Liver disease	1.52 (1.13–2.03)	1.06 (0.93–1.21)	1.29 (1.14–1.45)
Cancer	1.16 (0.97–1.39)	0.98 (0.90–1.07)	1.08 (0.99–1.18)
Diabetes	1.01 (0.90–1.14)	1.09 (1.03–1.14)	1.02 (0.96–1.08)
Hypertension	1.05 (0.91–1.20)	1.11 (1.05–1.17)	0.92 (0.86–0.98)
Dementia	0.90 (0.48–1.70)	0.69 (0.54–0.88)	0.84 (0.66–1.08)
HIV	0.80 (0.33–1.93)	1.13 (0.82–1.55)	0.93 (0.60–1.45)
Prior analyte value^b^			
	1 (Ref)	1 (Ref)	1 (Ref)
	1.22 (1.01–1.47)	1.18 (1.11–1.26)	1.54 (1.38–1.71)
	1.23 (1.02–1.50)	1.13 (1.06–1.20)	4.20 (3.81–4.63)
	1.26 (1.04–1.52)		
	0.96 (0.72–1.28)		

CKD, chronic kidney disease; COPD, chronic obstructive pulmonary disease; PTH, parathyroid hormone; Ref, reference.

a During 1-yr baseline.

b For PTH, 5 levels are as follows: 0–70 (ref), >70–110, >110–150, >150–300, >300 pg/ml; for 25D, 3 levels are as follows: ≥30 (ref), 20 to < 30, <20 ng/ml; for phosphorus, 3 levels are 1.5–4.5 (ref), >4.5–5.5, >5.5 mg/dl.

## Discussion

In this study, we used a large EHR database to examine patterns of testing, treatment, and retesting for CKD-MBD−related abnormalities in patients with non−dialysis-dependent CKD. We found, not unexpectedly given previous work from our group^4^ and others,^5^, ^6^, ^7^, ^8^, ^9^, ^10^, ^11^ that the frequency of testing appears to be suboptimal. However, we extend these findings by exploring the distributions of CKD-MBD analyte levels from initial testing, and the value at which physicians undertake treatment in actual clinical practice, the frequency of different CKD-MBD treatments, and the patterns of and factors associated with retesting following therapy initiation. We found times to analyte retesting following treatment initiation to be longer than might be expected, and, perhaps most unexpectedly, that pre-treatment levels of PTH and 25D were not associated with the rate of retesting for secondary hyperparathyroidism or 25D insufficiency, respectively.

We found that rates of test ordering increased across worsening CKD stages, although test ordering in general was far from universal. We were uncertain as to whether our results would be comparable to our previous results,^4^ given differences in the populations studied (a broad EHR population in the current study, versus Medicare beneficiaries only in our previous work) and in the ascertainment of CKD (combined use of ICD-9/10-CM diagnosis codes for CKD stage and confirmatory eGFR laboratory values in the present study, versus use of diagnosis codes exclusively in our previous work). Nevertheless, the percentages of testing of the various analytes were comparable: for example, compared with the 46% of stage 4 and 41% of stage 5 patients in the EHR database undergoing PTH testing in the current study, analogous rates in Medicare beneficiaries were 44% and 48%, respectively. Compared with the 74% of stage 4 and 73% of stage 5 patients undergoing a test for phosphorus in the present study, values for Medicare patients were 56% and 62%, respectively. Finally, compared with the 38% of stage 4 and 25% of stage 5 patients undergoing a test for 25D in the present study, respective values for Medicare beneficiaries were 39% and 29%. Because testing for PTH, phosphorus, and 25D likely is primarily undertaken by nephrologists, at least for stage 4 and 5 patients, this suggests that nephrologists may not be following the KDIGO guideline^3^ as much as would be desirable, potentially suggesting missed opportunities for CKD-MBD treatment.

Although our study provides important epidemiologic data on the distribution of levels of biochemical markers of CKD-MBD observed upon initial testing after CKD onset, a more important contribution might be the levels observed at the time patients are initiated on treatment. PTH levels before treatment were relatively high, at least according to historical standards. The most recent CKD-MBD KDIGO guideline^3^ is substantially less prescriptive than a previous iteration of the guideline, generally eschewing the recommendation of specific analyte levels that should trigger treatment. The latest guideline states that “in adult patients with CKD G3a-G5…we suggest calcitriol and vitamin D analogs not be routinely used,” and states that “it is reasonable to reserve the use of calcitriol and vitamin D analogs for patients with CKD G4-G5 with severe and progressive hyperparathyroidism.”^3^ We find evidence that treating physicians, possibly as a result of these guidelines, appear to be using relatively high treatment thresholds: mean levels before treatment for PTH were 154, 221, and 352 pg/ml, respectively, for stages 3, 4, and 5 disease. Whether this was the intended result of implementation of KDIGO guidelines should be discussed within the nephrology community, because the rise in levels of PTH, although initially adaptive, may well be maladaptive by the time the patients enters stage 5 or even stage 4 CKD. In contrast, insufficient 25D levels appear to have been treated more aggressively (mean levels >20 ng/ml at stages 3−5 upon initiation), although whether this is aggressive enough remains a matter of dispute.^13^^,^^14^ In the case of phosphorus, tolerance for hyperphosphatemia in stage 4 appeared to be low, with treatment beginning at a mean level of only 4.6 mg/dl, but levels that appeared to trigger treatment in stage 5 were much higher (mean of 5.8 mg/dl). The latter finding may not represent nihilism about treatment in stage 5 CKD, but might rather signify the many competing priorities that nephrologist face in CKD stage 5, such as preparation for dialysis, creation of a vascular access, treatment of anemia, and so on.

The KDIGO guideline provides recommendations for the frequency of monitoring for development and progression of CKD-MBD−related abnormalities, but these might not be meant for patients actually receiving treatment. It seems reasonable that providers would test patients receiving treatment at least as frequently as patients being monitored for the potential development of CKD-MBD−related abnormalities. In the case of PTH, KDIGO recommends retesting at 6 to 12 months for stage 4 and at 3 to 6 months for stage 5 disease (with no recommendations for stage 3). Although half of patients underwent retesting for PTH by approximately 8 months for stage 4 and by 4.5 months for stage 5, the proportion retested appeared to plateau at approximately 50% to 55% by 1 year for patients in both groups, suggesting that a substantial number of patients were not being retested. The proportion of patients undergoing retesting for phosphorus, which is recommended every 6 to 12 months for CKD stage 3 patients, 3 to 6 months for CKD stage 4 patients, and 1 to 3 months for CKD stage 5 patients, rapidly reached 50% (by approximately 2 months for stages 4 and 5 and by 4 months for stage 3), but again tended to plateau at 50% to 60% for all stages by about 6 months, suggesting that a substantial subset of patients do not appear to be retested despite treatment. Unless some testing is occurring outside of the EHR system, we cannot be certain as to the specific reasons for the apparent low rate of retesting. One possible explanation may be uncertainty over the cost-effectiveness of repeated testing after treatment. Although it makes clinical sense to retest in order to assess response (or lack thereof) to a particular therapy, the cost-effectiveness of such an action has not been evaluated in terms of hard outcomes. In addition, because the guidelines generally focus on pretreatment detection of disease, providers may believe that the guidelines do not offer adequate direction for the frequency or even the necessity of post−treatment-initiation testing. This is merely a hypothesis, however, the specific exploration of which we did not attempt to undertake.

Perhaps most unexpectedly, only in the case of phosphorus were higher pretreatment levels associated in a clear dose-dependent manner with an increased rate of retesting; for both PTH and 25D, this type of dose−response relationship was lacking. The KDIGO guideline states that the “frequency of monitoring” should be based on the “presence and magnitude of abnormalities.^3^“ We did not find evidence that this was the case for PTH or indeed for 25D (although KDIGO does not consider 25D testing specifically).

Several clinically relevant factors were associated with increased or decreased hazard ratios (HRs) for analyte testing. Patients with liver disease had significantly increased HRs for testing for PTH and P (and a point estimate >1 for testing for 25D). This could reflect providers’ specific concerns about nutritional status in patients with liver disease. Patients with dementia, in contrast, had point estimates <1 for testing for all 3 analytes (although none were statistically “significant”); this may indicate that the testing for these analytes in patients with dementia and presumably frailty is considered superfluous. One additional factor associated with decreased re-testing for PTH and 25D is a history of hospitalization in the previous year. It is likely that a history of relatively recent hospitalization is a general marker for illness, particularly acute or subacute illness. As such, concerns about appropriate treatment for 25D deficiency or SHPT might be viewed as secondary in importance by providers, who presumably are attempting to address competing medical issues by adjusting other medications of more immediate perceived importance. If true, this finding, albeit perhaps understandable, is unfortunate, because control of CKD-MBD abnormalities is likely no less important in sicker patients than in healthier ones and may indeed be more so.

Our study is subject to some important limitations. First, stronger inferences about testing and treatment can be made for PTH and 25D than for calcium, phosphorus, and ALP, because the former 2 analytes required dedicated tests, whereas the latter 3 are typically ordered as elements of a panel, such as basic metabolic or renal panels. Second, in an observational study such as ours, we cannot determine causality, only temporal antecedence; we therefore cannot claim that the value of an analyte caused or prompted a physician to initiate treatment. Third, assessment of treatment for hyperphosphatemia was limited by the fact that patients might often use over-the-counter calcium carbonate, which cannot be ascertained in the database that we used.

In summary, by using a large EHR database containing laboratory data, we found that rates of testing for CKD-MBD−related abnormalities appear to be lower than advocated by the KDIGO guideline. Levels of PTH measured immediately before commencement of treatment appear to be relatively high, which may be due to physicians practicing under the current KDIGO guideline, which are less prescriptive than some previous guidelines.^15^ Retesting of biochemical markers after treatment initiation did not appear to occur in a timely fashion (i.e., within at least 1 year) for a large fraction of patients. Paradoxically, for the case of PTH and 25D, although not for phosphorus, the pretreatment level of the analyte was not associated in a dose-dependent manner with the rate of retesting following the initiation of therapy. How the KDIGO guideline is being implemented in actual clinical practice should be discussed by the community.

## Disclosure

AA is an employee of OPKO Health, Inc., which supported the study. The sponsor played a role in the study design, but had no role in the decision to publish the manuscript. All the other authors declared no competing interests.

## Supplementary Material

Supplementary File (PDF)

**Table S1.** EHR codes used for defining the CKD cohorts

**Table S2.** EHR codes used to identify testing and lab results related to monitoring for CKD-MBD abnormalities

**Table S3.** RxNorm codes used to identify medications prescribed for CKD-MBD abnormalities

**Table S4.** ICD-9/10-CM codes used to identify comorbid conditions

**Table S5.** Follow-up time in the CKD stage 3, 4, and 5 cohorts

**Figure S1.** Density plots displaying the full distribution of lab results for each biochemical marker.
