# Supplementary material for: Testing Patterns for CKD-MBD Abnormalities in a Sample US Population
Source: Kidney Int Rep. 2021 Jan 6;6(4):1141–50. doi: 10.1016/j.ekir.2020.12.036 (PMC8071625; doi:10.1016/j.ekir.2020.12.036)
Supplement: Supplementary File (PDF) [file mmc1.pdf]

Supplementary Table S1. EHR codes used for defining the CKD cohorts

CKD Diagnosis (SNOMED CT)		
433144002 (stage 3)		
431857002 (stage 4)		
433146000 (stage 5)		
eGFR Lab Result (LOINC)		
69405-9	50044-7	70969-1
33914-3	48642-3	88293-6
62238-1	77147-7	88294-4
48643-1	50210-4	76633-7
Creatinine Lab Result* (LOINC)		
77140-2	2160-0	35203-9
59826-8	14682-9	45066-8
38483-4	72271-0	
ESRD or Kidney Transplant (SNOMED CT)		
251869004	428982002	238321006
105502003	698810000	398887003
236435004	46177005	70536003
443596009	67970008	23137002
33461007	238318009	175902000
442326005	238319001	
70536003	71192002	

*When a creatinine value was available in the EHR, but there was no corresponding eGFR value, we calculated eGFR using the MDRD equation (Levey Ann Intern Med 2006;145(4):247-254).

CKD, chronic kidney disease; eGFR, estimated glomerular filtration rate; EHR, electronic health rcord; LOINC: Logical Information Identifiers Names and Codes, SNOMED CT: Systematized Nomenclature of Medicine Clinical Terms.

Supplementary Table S2. EHR codes used to identify testing and lab results related to monitoring for CKD-MBD abnormalities

Testing (SNOMED CT and CPT)		
PTH SNOMED CT:<br>3352000<br>169181004<br>271541003<br>391554001		PTH CPT:<br>83970<br>3278F
Phosphorus SNOMED CT:<br>8364005<br>104866001<br>121797002<br>312473009	Phosphorus SNOMED CT:<br>271241002<br>401145005<br>104868000<br>44277000	Phosphorus CPT<br>84100<br>80069<br>3278F
25D SNOMED CT: 83729008<br>104656006<br>169203007<br>270990000<br>447680002<br>412925008	25D SNOMED CT:<br>12199005<br>447678008<br>313756002<br>313757006<br>105029001<br>447679000	25D CPT:<br>82306<br>82307
Calcium SNOMED CT:<br>71878006<br>312472004<br>711359007<br>390963002<br>390966005<br>271240001<br>166708003<br>391084000	Calcium SNOMED CT:<br>391551009<br>443796007<br>442156005<br>252148000<br>167209002<br>44277000<br>165102003	Calcium CPT:<br>82310<br>82330<br>80047<br>80048<br>80053<br>80069<br>3278F
ALP SNOMED CT:<br>88810008<br>8244002<br>271052004<br>313846006<br>313845005	ALP SNOMED CT:<br>390962007<br>271234008<br>167209002<br>26958001	ALP CPT:<br>84075<br>84080<br>80053<br>80076
Lab result (LOINC)		
PTH: 2731-8, 14866-8 (*excluding values > 6000 pg/mL*)	25D: 1989-3, 62292-8, 14635-7 (*excluding values >200 ng/mL*)	ALP: 6768-6, 1783-0 (*excluding values < 20 or > 500 U/L*)
Phosphorus: 2777-1, 2774-8, 14879-1, 24519-1 (*excluding values < 1.5 or > 16 mg/dL*)	Calcium: 17861-6, 49765-1, 2000-8, 1996-8 (*excluding values <6 or >18 mg/dL*)	

ALP, alkaline phosphatase; CKD-MBD, chronic kidney disease mineral and bone disorder; CPT, Current Procedural Terminology; LOINC: Logical Information Identifiers Names and Codes; PTH, parathyroid hormone; SNOMED CT: Systematized Nomenclature of Medicine Clinical Terms.

Supplementary Table S3. RxNorm codes used to identify medications prescribed for CKD-MBD abnormalities

Activated vitamin D compounds			
Calcitriol:			
1894	261283		
152323	308867		
209006	308868		
209007	313932		
Paricalcitol:			
73710	577324		
221005	606854		
577315	606859		
577317	606861		
Doxercalciferol:			
11516	406503		
261247	540687		
261539	858262		
310023	858264		
Calcifediol:			
1889			
1855066			
1855072			
Nutritional vitamin D			
Ergocalciferol: 4018,			
92515, 107263	259870, 310142	1007511, 1007512	1367409, 1367410
197645, 199832	485550, 654203	1007516, 1008264	1367416, 1367421
212374, 237402	795480, 877408	1014182, 1086605	1941742, 2044659
237723, 245493	991456, 1001409	1088798, 1100896	
245494	1001758, 1007285	1112493, 1233664	
Cholecalciferol:			
2418, 92066	833746, 835531	1091242, 1091245	1292776, 1297389
150923, 199362	845523, 846138	1091283, 1092183	1298198, 1298199
204634, 209638	846146, 847121	1092380, 1094022	1299811, 1302680
210133, 215818	849410, 849530	1095603, 1095605,	1303911, 1303916
215819, 216438	857466, 861054	1095608, 1098884	1304498, 1304503
240029, 245500	864665, 864669	1098887, 1099861	1305135, 1310948
248506, 251154	865078, 865087	1099869, 1100262	1310954, 1312502
309263, 360422	866406, 877318	1100466, 1100469	1313925, 1313928
428485, 435020	880340, 881314	1100894, 1111681	1313933, 1362805
485689, 577055	884773, 885489	1114604, 1114846	1365980, 1366144
577068, 607553	891692, 898694	1114847, 1115497	1366149, 1370012
608343, 608349	904462, 904465	1115877, 1116508	1370013, 1372696
616551, 644254	904492, 904495	116511, 1116514	1375985, 1424840

644256, 668292	967916, 967919	1119570, 1119573	1424841, 1425205
685054, 701441	991376, 994794	1125499, 1125500	1429287, 1432986
705726, 705728	994831, 1001659	1148597, 1148599	1435912, 1435917
707647, 724165	1001679, 1001691	1150266, 1189592	1437783, 1442442
724411, 762908	1001751, 1005702	1189599, 1193036	1442443, 1485531
801663, 802795	1006914, 1007068	1193037, 1193041	1486349, 1488353
804146, 804614	1007121, 1007378	1193046, 1233031	1493427, 1536164
804617, 808596	1007560, 1007763	1233032, 1233607	1543212, 1593743
809533, 809536	1007835, 1008035	1234482, 1243353	1593744, 1601102
809540, 809555	1008292, 1008522	1243364, 1243393	1650274, 1651279
809556, 809559	1008555, 1008761	1243399, 1243405	1651282, 1720258
809572, 828296	1008768, 1008792	1243415, 1244008	1741416, 1746494
829749, 829758	1008800, 1041795	1244014, 1244021	1789542, 1790877
829759, 829993	1042692, 1043300	1244924, 1244925	1809754, 1870970
829998, 832907	1043301, 1087280	1244926, 1244930	1874857, 1919489
832908, 832909	1087282, 1087285	1244936, 1246098	1996206
832910, 832919	1088134, 1088432	1291265, 1291276	
832924, 833456	1088534, 1088800	1291595, 1292029	
833741			
Oral phosphate binders			
Sevelamer: 135118	749207, 749210	857224, 857226	
214824, 237125	857208, 857210	861370, 861373	
660890, 749206	857216, 857218	861375, 861377	
Calcium acetate:			
197433, 200147	215849, 219236	645216, 830639	
209010, 209011	245133, 282539	830642, 1099804	
214342, 214344	359296, 428703	1099805, 1099808	
Calcium carbonate:			
1897, 91254,	809559, 809572	359297, 360422	1092183, 1094022
92066, 150923,	817958, 821119	384518, 403826	1099861, 1099869
171000, 198527	824590, 826091	403828, 404179	1099878, 1100466
198529, 198530	828296, 829758	428485, 428633	1100894, 1100896
198531, 198533	829759, 832907	428634, 476549	1102202, 1102207
198534, 198535	832908, 832909	577040, 577049	1111681, 1112493
198542, 198864	832910, 832919	577181, 606683	1112810, 1114604
198865, 199296	832924, 833456	607278, 608343	1125499, 1125500
200091, 200154	833741, 833746	642335, 642338	1189592, 1189599
200189, 200350	845523, 861054	642355, 642356	1233607, 1243353
204634, 204636	864665, 864669	644254, 645048	1243364, 1243393
205075, 205233	865078, 865087	647558, 654203	1243399, 1243405
210097, 210116	866486, 867494	655935, 668292	1243415, 1244021
210117, 210133	867497, 884773	688892, 691005	1244924, 1244925
210135, 210143	905095, 991370	691006, 691010	1244926, 1244930
210248, 210249	991376, 994240	691012, 701441	1244936, 1245383
212460, 212462	994243, 994794	706946, 707270	1245384, 1245389
212495, 212825	994831, 998704	707558, 729490	1245406, 1248142
214346, 214347	1001409, 1001659	790271, 794747	1251312, 1297389
214349, 215817	1001679, 1001691	795182, 795186,	1298165, 1299811

215818, 215822<br>215829 215830,<br>215835 215850,<br>218833 218957,<br>218959 218991,<br>218995 219327,<br>220327 220508,<br>220509<br>220510, 220511<br>237402, 237420<br>237865, 237867<br>242623, 250222<br>259285, 259567<br>259590, 261616<br>261688, 282465<br>282746, 283174<br>809540, 809547<br>809555, 809556	1006914, 1007068<br>1007166, 1007378<br>1007404, 1007498<br>1007511, 1007763<br>283641, 284051<br>307685, 308844<br>308888, 308889<br>308891, 308892<br>308895, 308896<br>308900, 308903<br>308905, 308906<br>308907, 308914<br>308915, 313884<br>315015, 317865<br>318076, 346210<br>346211, 351590<br>352490, 352491	97654, 808596,<br>09533, 809536<br>1007775, 1007778<br>1008004, 1008237<br>1008264, 1008515<br>1008522, 1008555<br>1008556, 1008623<br>1008749, 1008800<br>1009020, 1009033<br>1041833, 1044524<br>1044532, 1086605<br>1087258, 1087259<br>1087262, 1087280<br>1087282, 1087285<br>1088798, 1088800<br>1088805, 1091242<br>1091245, 1091283	1302636, 1302637<br>1310948, 1310954<br>1311062, 1313925<br>1362805, 1367409<br>1367421, 1372696<br>1426128, 1432465<br>1432529, 1437783<br>1438105, 1441385<br>1442442, 1442443<br>1484731, 1491617<br>1491618, 1492387<br>1493427, 1536164<br>1538595, 1540539<br>1543212, 1607303<br>1653049, 1722689<br>1722690, 1722695<br>1789542
Lanthanum carbonate:<br>234416, 356761<br>477347, 542465	602635, 603112<br>603122, 642452<br>1551881, 1551884<br>1551886		
Sucroferric oxyhydroxide:<br>1484283, 1484289<br>1484290, 1484296			
Ferric citrate:<br>1594675, 1594680<br>1596018, 1596023			

CKD-MBD, chronic kidney disease mineral bone disorder,

Supplementary Table S4. ICD-9/10-CM codes used to identify comorbid conditions.

Atherosclerotic heart disease			
410	414	I21	I25
411	V45.81	I22	Z95.1
412	V45.82	I23	Z95.5
413	I20	I24	Z98.61

Congestive heart failure			
398.91	404.91	I09.9	I42.7
402.01	404.93	I11.0	I42.8
402.11	425.4	I13.0	I42.9
402.91	425.5	I13.2	I43
404.01	425.7	I25.5	I50
404.03	425.8	I42.0	P29.0
404.11	425.9	I42.5	
404.13	428	I42.6	

Cardiac arrhythmia			
426.0	427.2	996.04	I47
426.13	427.3	V45.0	I48
426.7	427.4	V53.3	I49
426.9	427.6	I44.1	R00.0
426.10	427.8	I44.2	R00.1
426.12	427.9	I44.3	R00.8
427.0	785.0	I45.6	T82.1
427.1,	996.01	I45.9	Z45.0
			Z95.0

Cerebrovascular disease			
362.34	435	I60	I66
430	436	I61	I67
431	437	I62	I68
432	438	I63	I69
433	G45	I64	H34.0
434	G46	I65	

Peripheral vascular disease			
093.0	443.8	I70	I77.1
440	443.9	I71	I79.0
441	447.1	I73.1	I79.2
443.1	557	I73.8	K55
443.2	V43.4	I73.9	Z95.82

Chronic obstructive pulmonary disease			
416.8	502	J42	J65
416.9	503	J43	J66
490	504	J44	J67
491	505	J45	J68.4

492	506.4	J46	J70.1
493	508.1	J47	J70.3
494	508.8	J60	
495	I27.8	J61	
496	I27.9	J62	
500	J40	J63	
501	J41	J64	
Gastrointestinal bleeding			
456.0, 456.20,	533.01, 533.10	535.51, 535.61	K27.6, K28.0
530.21, 531.00	533.11, 533.20	535.71, 537.83	K28.1, K28.2
531.01, 531.10	533.21, 533.40	537.84, 569.85	K28.4, K28.5
531.11, 531.20	533.41, 533.50	578.0, 578.9	K28.6, K29.01
531.21, 531.40	533.51, 533.60	I85.01, I85.11	K29.21, K29.31
531.41, 531.50	533.61, 534.00	K22.11, K25.0	K29.41, K29.51
531.51, 531.60	534.01, 534.10	K25.1, K25.2	K29.61, K29.71
531.61, 532.00	534.11, 534.20	K25.4, K25.5	K29.81, K29.91
532.01, 532.10	534.21, 534.40	K25.6, K26.0	K31.811, K31.82
532.11, 532.20	534.41, 534.50	K26.1, K26.2	K55.21, K92.0
532.21, 532.40	534.51, 534.60	K26.4, K26.5	K92.2
532.41, 532.50	534.61, 535.01	K26.6, K27.0	
532.51, 532.60	535.11, 535.21	K27.1, K27.2	
532.61, 533.00	535.31, 535.41	K27.4, K27.5	
Liver disease			
070.22, 070.23	570, 571, 572.2	B19.20, B19.21,	K76.0, K76.2,
070.32, 070.33	572.3, 572.4	B19.9, I85, I86.4,	K76.3, K76.4,
070.44, 070.54	572.8, 573.3	I98.2, K70, K71.1,	K76.5, K76.6,
070.6, 070.7	573.4, 573.8	K71.3, K71.4,	K76.7, K76.8,
070.9, 456.0	573.9, V42.7	K71.5, K71.7,	K76.9, Z94.4
456.1, 456.2	B18, B19.0	K72, K73, K74,	
Cancer			
196, 197, 198	C62, C63, C64	C74, C75, C76	C08, C09, C10
199, 140, 141	160, 161, 162	C97, 188, 189	C11, C12, C13
142, 143, 144	163, 164, 165	190, 191, 192	C14, C15, C16
145, 146, 147	166, 167, 168	193, 194, 195	C17, C18, C19
148, 149, 150	169, 170, 171	200, 201, 202	C20, C21, C22
151, 152, 153	172, 174, 175	203.0, 238.6	C23, C24, C25
154, 155, 156	176, 177, 178	C81, C82, C83	C26, C30, C31
157, 158, 159	179, 180, 181	C84, C85, C88	C32, C33, C34
C46, C47, C48	182, 183, 184	C96, C90.0	C37, C38, C39
C49, C50, C51	185, 186, 187	C90.2, C77, C78	C40, C41, C43
C52, C53, C54	C65, C66, C67	C79, C80, C00	C45
C55, C56, C57	C68, C69, C70	C01, C02, C03	
C58, C60, C61	C71, C72, C73	C04, C05, C06	
		C07	
Diabetes			
250	E11		

E10	E13	
Hypertension		
401	405	I13
402	I10	I15
403	I11	I16
404	I12	
Dementia		
290	F02	
F00	F03	
F01	G30	
HIV		
042	V08	B22
043	B20	B24
044	B21	Z21

ICD-9/10-CM, International Classification of Diseases, Ninth/Tenth Revision, Clinical Modification.

Supplementary Table S5. Follow-up time in the CKD stage 3, 4 and 5 cohorts

	CKD stage 3	CKD stage 4	CKD stage 5
	n = 215,553	*n* = 43,576	*n* = 11,407
Follow up time, days			
Mean (SD)	838.84 (634.36)	603.82 (546.74)	219.45 (320.95)
Median (25th, 75th)	690 (382, 1165)	457 (187, 847)	81 (21 ,309)
Follow up time, years			
Mean (SD)	2.30 (1.74)	1.65 (1.50)	0.60 (0.88)
Median (25th, 75th)	1.89 (1.05, 3.19)	1.25 (0.51, 2.32)	0.22 (0.06, 0.85)

CKD, chronic kidney disease; SD, standard deviation.

Figure S1. Density plots displaying the full distribution of lab results for each biochemical marker.

PTH

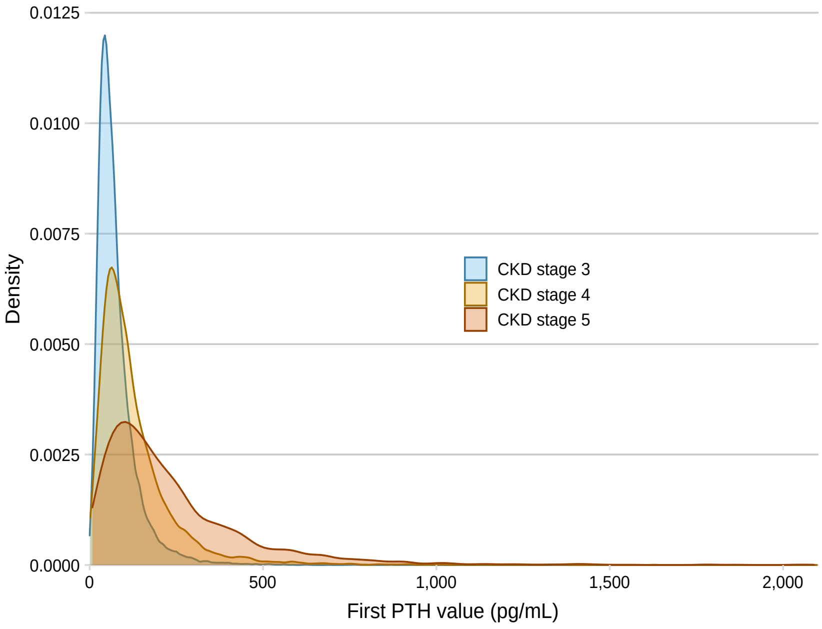

25D

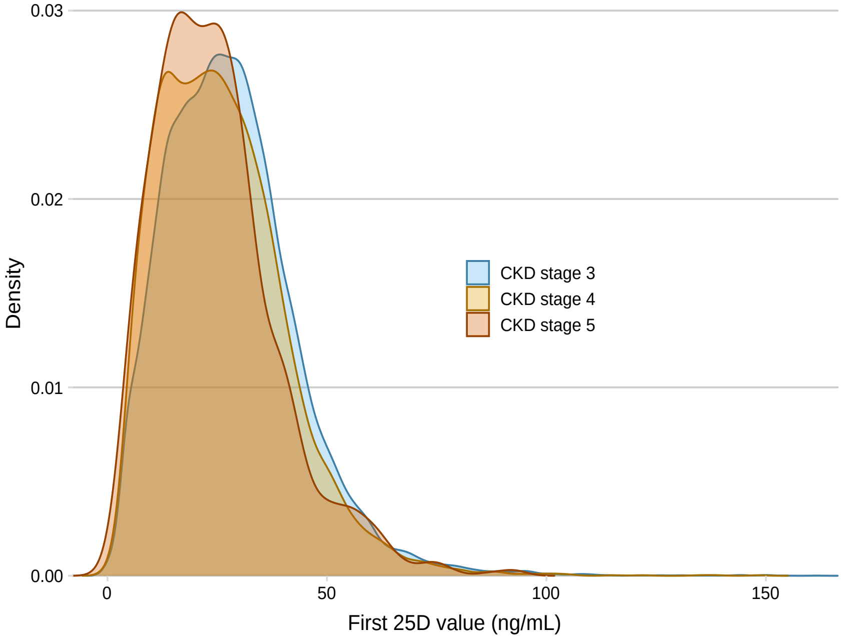

Phosphorus

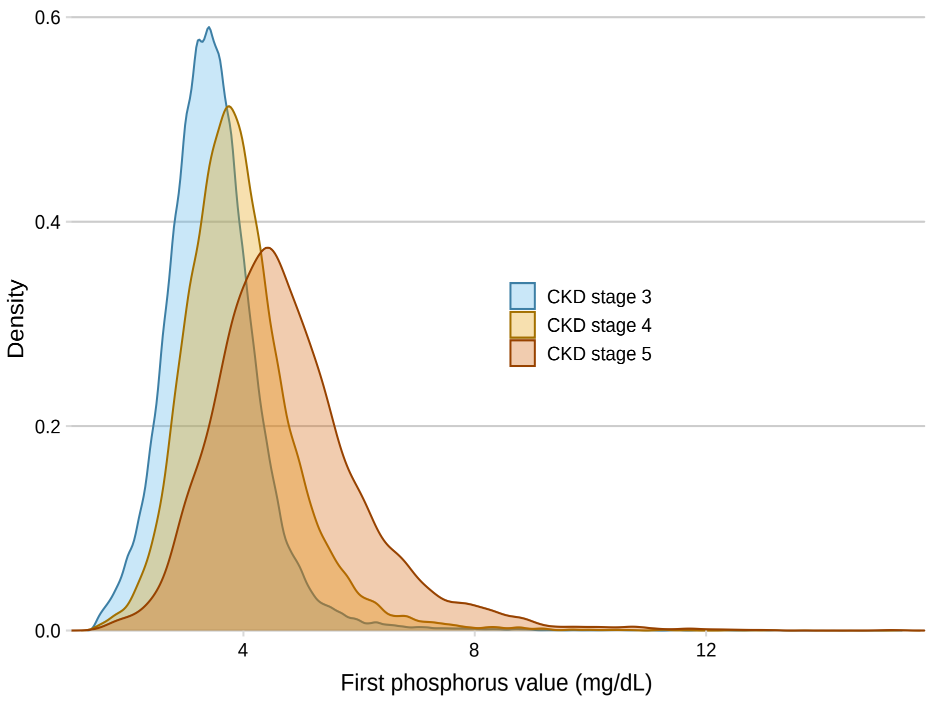

0.6
0.4
0.2
0.0
Density
CKD stage 3
CKD stage 4
CKD stage 5
4
8
12
First phosphorus value (mg/dL)
